# Alexander Friedenstein, Mesenchymal Stem Cells, Shifting Paradigms and Euphemisms

**DOI:** 10.3390/bioengineering11060534

**Published:** 2024-05-23

**Authors:** Donald G. Phinney

**Affiliations:** Department of Molecular Medicine, Herbert Wertheim UF Scripps Institute for Biomedical Innovation and Technology, Jupiter, FL 33458, USA; dphinney@ufl.edu

**Keywords:** mesenchymal stem cells, mesenchymal stromal cells, stromal cells, stem cells, paracrine communication, hierarchy

## Abstract

Six decades ago, Friedenstein and coworkers published a series of seminal papers identifying a cell population in bone marrow with osteogenic potential, now referred to as mesenchymal stem cells (MSCs). This work was also instrumental in establishing the identity of hematopoietic stem cell and the identification of skeletal stem/progenitor cell (SSPC) populations in various skeletal compartments. In recognition of the centenary year of Friedenstein’s birth, I review key aspects of his work and discuss the evolving concept of the MSC and its various euphemisms indorsed by changing paradigms in the field. I also discuss the recent emphasis on MSC stromal quality attributes and how emerging data demonstrating a mechanistic link between stromal and stem/progenitor functions bring renewed relevance to Friedenstein’s contributions and much needed unity to the field.

## 1. Friedenstein, Transitional Epithelium, and Diffusion Chambers

**Figure 1:**
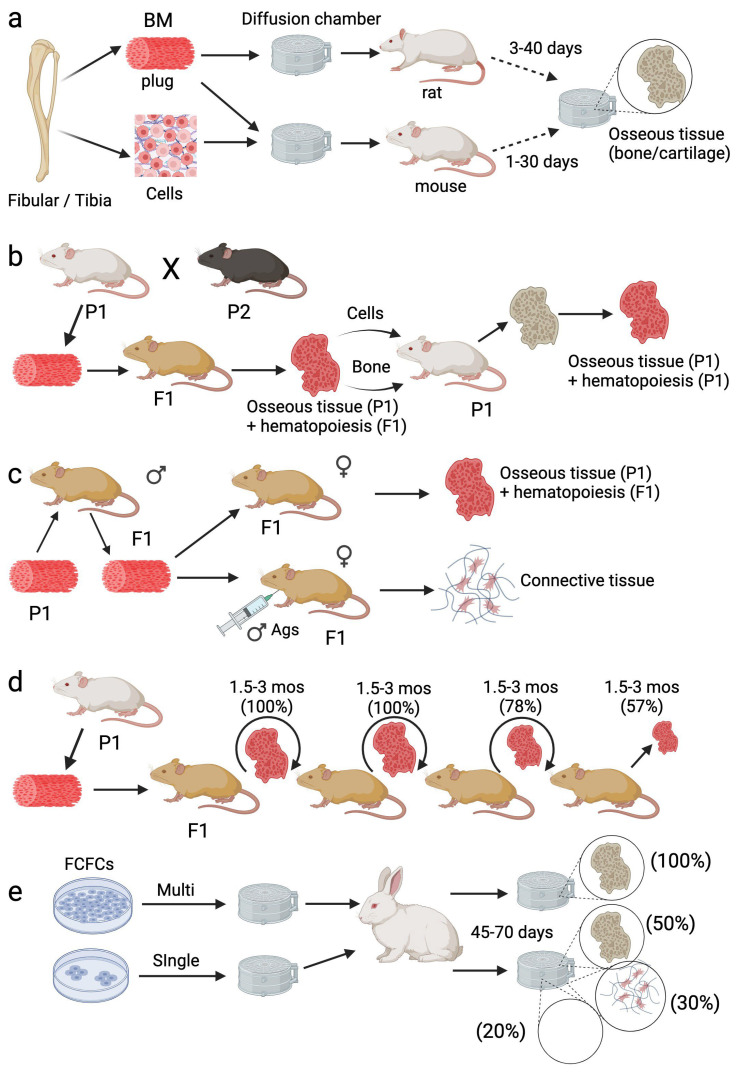
Experimental paradigms to establish the existence of marrow resident osteogenic stem cells. (**a**) Bone marrow plug or cell suspension was encapsulated in diffusion chambers, transplanted into the peritoneal cavity of rats or mice, and, at the indicated time points, recovered and analyzed for bone formation. (**b**) Bone marrow plug from a parental strain (P1) was transplanted into an F1 hybrid, recovered at 19–60 days, 3–6 months, or 12–14 months, and analyzed for bone tissue. Bone or bone marrow kept in F1 hybrids for various durations (up to 14 months) was retransplanted back into the P1 strain and analyzed at 6–15 days or 28–60 days post-transplant for bone formation. (**c**) Bone marrow plug from male mice was transplanted under the renal capsule of naïve female mice or female mice immunized with male antigens and bone formation analyzed at 30–40 days post-transplant. (**d**) Bone marrow plug from P1 mice was grafted under the renal capsule of F1 mice, and every 1.5–3 months, bone marrow blown out of the heterotopic ossicle was regrafted under the renal capsule of a new F1 recipient. (**e**), Single or multiple colonies of adhesive fibroblasts were obtained by explanting bone marrow from the pelvic bone of rabbits, encapsulated in diffusion chambers, and transplanted into rabbits. Chambers were recovered at 45–70 days post-transplant and analyzed for bone formation. Created with BioRender.com.

Friedenstein’s early research focused on the osteo-inductive properties of transitional epithelium. Specifically, he showed that osteogenesis was stimulated in connective tissue in direct contact with undifferentiated epithelial cells; these contact regions were rich in glycogen, and that immunological rejection of the epithelium resulted in the resorption of ectopic bone and its replacement with connective tissue [1]. He further showed that subcutaneous transplants of urinary bladder mucosa encapsulated in diffusion chambers stimulated ectopic bone formation, indicating that the former secretes a substance that stimulates bone formation at a distance [2]. Perhaps inspired by the work of Rosin et al. [3], Friedenstein subsequently transplanted tibial bone marrow encapsulated in diffusion chambers into the peritoneal cavity of rats [4] and observed foci of osteogenesis originating from large, stellate reticular cells by 3 weeks post-transplant, followed by deposition of bone and cartilage, thereby revealing the bipotential nature of marrow resident precursor cells (Figure 1a). Related studies employing fragments or cell suspensions of femoral bone marrow yielded similar findings, confirming that osteogenic activity was not lost by the dissociation of bone marrow plugs. Using chambers of different sizes, Friedenstein also showed that a cell density threshold existed to achieve osteogenesis, and cells that gave rise to osteogenic foci possessed high mitotic activity. Based on these findings, he postulated the existence of separate stem cells for hematopoietic and stromal elements in bone marrow and that bone and reticular (stromal) cells likely share a common origin.

## 2. Evidence for a Bone Marrow Osteogenic Stem Cell

To identify the origin of the marrow resident osteogenic precursor, Friedenstein conducted a series of syngeneic, semi-syngeneic, allogeneic, and reverse transplants in mice [5]. Herein, bone marrow from a parental strain grafted under the kidney capsule of a syngeneic or F1 hybrid (semi-syngeneic) recipient generated well-developed bone tissue that remained viable beyond one-year post-transplant and supported active hematopoiesis. Karyotype analysis confirmed that bone tissue formed in semi-syngeneic transplants originated from parental donor cells, while hematopoietic cells colonizing the bone derived from the transplant recipients (Figure 1b). When bone marrow was transplanted back into the original donor strain, hematopoiesis ceased by the end of the first week and recommenced several weeks later, indicative of the replacement of the F1 cells with those from the parental transplant recipient. Over time, these transplants developed into osseous organs with actively proliferating bone marrow, which increased in volume, although several of the retransplants were absorbed. Alternatively, retransplanted bone tissue was not subject to resorption irrespective of the time it remained in the original recipient, which ranged from 42 days to 14 months, and therefore behaved as a syngeneic transplant. These results contrasted sharply with that obtained from allogeneic transplants, wherein a brief period of osteogenesis was observed that was devoid of hematopoiesis, followed by an active period of resorption such that by 40 days post-transplant, most grafts showed no traces of bone and/or contained small remnants of dead bone infiltrated by lymphocytes.

While the results from the reverse transplantation experiments were compelling, they fell short of demonstrating the exact nature of the osteogenic precursor. Therefore, Friedenstein conducted additional studies wherein bone marrow from a parental strain of female mice was transplanted into an F1 hybrid strain and then retransplanted into native F1 males or those pre-immunized against female antigens (Figure 1c) [6]. The results obtained in non-immunized recipients were like that observed in syngeneic transplants, while transplants conducted in pre-immunized recipients yielded outcomes akin to allogeneic transplants, i.e., immunological rejection of donor cells prevented ectopic bone formation. These results demonstrated that the osteogenic precursors present in bone marrow derive from the original donor strain and not from that of the transplant recipient. Based on data showing heterotopic osseous tissue from a single semi-syngeneic transplant was maintained for up to 14 months in vivo, Friedenstein then conducted serial transplants to interrogate the capacity of these precursors to self-renew [6]. Bone marrow recovered from heterotopic ossicles at 1.5–3 months post-transplant was regrafted under the renal capsule of new F1 recipients, and in some cases, the marrow cells were simultaneously transplanted back into the original parental strain. The results from these studies showed that the osteogenic capacity of marrow cells was preserved after two serial transplants, e.g., 100% (9/9) of transplants yielded osseous tissue, and this was reduced to 78% (7/9) on the third transplant and 57% (4/7) by the fourth transplant (Figure 1d). Since the amount of bone tissue generated on the fourth transplant was too minimal to support another serial passage, Friedesntein concluded that osteogenic capacity was exhausted by the fourth passage. These results paralleled independent data showing that the capacity of bone marrow to regenerate the hematopoietic system was exhausted after 3–5 passages through irradiated recipients. 

In related studies, Friedenstein quantified the osteogenic potential of bone marrow obtained from irradiated donors grafted into non-irradiated syngeneic and semi-syngeneic recipients [6]. This capacity was retained in cells transplanted at 2 h but not 4–10 days post-exposure to 825 rad and was not evident at 2 h post-transplant in cells obtained from mice exposed to >3000 rad. Importantly, osteogenic activity was restored after 1 month post-irradiation when bone marrow was transplanted in recipient mice but not the irradiated donor. Systemic infusion of hematopoietic cells into irradiated donors did not restore osteogenic activity. The results from these studies indicated that bone marrow contains a low frequency of cells with osteogenic potential that are radio-resistant compared to their hematopoietic counterparts. 

## 3. Fibroblast Colony-Forming Cells (FCFCs), Colony-Forming Unit-Fibroblasts (CFU-Fs), and Multi-Potency

Having established that osteogenic precursors are unique, capable of self-maintenance, and regenerate independently of hematopoietic cells, Friedenstein pursued their characterization. Specifically, he showed that explant cultures of guinea pig bone marrow consisted initially of neutrophils and monocytes/macrophages that were gradually replaced by fibroblast colonies whose abundance was proportional to the number of explanted marrow cells [7]. Metaphase spreads of mixed cultures from male and female bone marrow donors showed fibroblastic colonies derived from a single donor, indicative of their clonal nature. Subsequently, he demonstrated that fibroblast colony-forming cells from guinea pig, rabbit, and human bone marrow were highly adhesive, possessed a high proliferative potential, could be maintained in culture for up to 15 passages, and those from bone marrow but not spleen generated heterotopic osseous tissue when inoculated into diffusion chambers and implanted in vivo (Figure 1e) [7]. This activity was also shown to be enhanced in the presence of transitional epithelium. Based on these results, Friedenstein postulated that FCFCs are marrow stromal cell precursors and responsible for transferring the microenvironment typical of hematopoietic tissue [8]. He further reported that the osteogenic activity of FCFCs was retained after 18 passages in vitro, and that the number of osteogenic precursors in culture increased significantly as a function of passage, indicating that several FCFCs have the potential to form large amounts (kilograms) of bone tissue. In related studies, he showed that cultures derived from single FCFCs possessed variable osteogenic activity when encapsulated in diffusion chambers and grafted in vivo (~50% yielded bone and/or cartilage, ~30% yielded connective tissue, and ~20% were devoid of cells), but the bone tissue formed was comparable in quality to that generated by multi-colony FCFCs (Figure 1e) [9]. Lastly, Friedenstein investigated the growth factor requirements for FCFC formation, identifying PDGF, FGF2, EGF, and TGF-beta as playing important but distinct roles in the growth of mouse vs. human colonies [10]. 

Friedenstein’s experimental results were foundational to our current understanding of the MSC. Various groups subsequently validated key observations regarding the functional heterogeneity and self-renewing capability of FCFCs, more commonly referred to as CFU-Fs. For example, using clone-splitting assays, Russell et al. [11] showed that CFU-Fs generated from two parental human MSC donor populations contained all eight possible categories of potency, which extended findings from other groups [12,13,14,15,16], and that the growth and death rates of clones were proportional to their potency, with tri-potent clones proliferating the fastest and uni-potent clones exhibiting the highest apoptosis rates [17]. Tri-potent clones were also shown to retain potency over at least three passages in vitro. Independent studies by Colter et al. [18,19] demonstrated that MSCs plated at low density exhibited the highest proliferative potential, expanding 2000-fold over 10 days, and these cultures consisted of distinct cell subsets, including small, agranular RS1 cells, small, granular RS-2 cells, and large, moderately granular mMSCs based on flow cytometric analysis. The authors further showed that RS-1 cells generated RS-2 cells during lag phase growth, RS-1 cells yielded mMSCs during log phase growth, RS-2 cells regenerated RS-1 cells during late log phase, and RS-1 cells reinitiated the cycle after replating, thereby maintaining the multi-potency of populations. In the 1980s, Owen and Friedenstein proposed using lineage tracing with genetic markers to verify the stem cell nature of marrow resident osteogenic precursors. As anticipated, this approach successfully identified several candidate skeletal stem/progenitor cell (SSPC) populations, including *Nestin^+^* [20] and *LepR^+^* [21] cells, while more recent studies combining lineage tracing with single cell RNA-based sequencing identified additional SSPCs in bone marrow, the growth plate, the periosteum, and calvarial sutures [22]. It is also well established that SSPCs resident in bone marrow secrete cytokines/chemokines that govern the retention and maintenance of HSCs [23]. These findings validate Friedenstein’s concept of the osteogenic stem cell, which was operationally correct but has since evolved to include a multitude of unique (but likely overlapping) entities within distinct skeletal compartments that function in the development, remodeling, and repair of bone tissue. In this regard, the skeletal compartment now appears to be more complex than its hematopoietic counterpart. 

## 4. Euphemisms and Paradigms 

**Figure 2:**
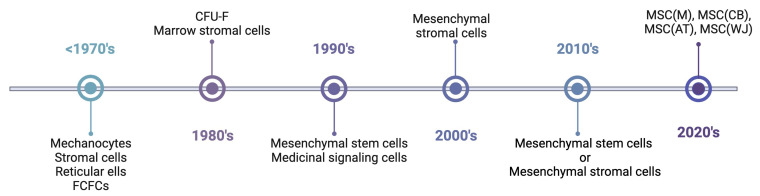
MSC nomenclature timeline. The timeline shows approximate periods when specific terminology was widely used in the literature. The timeline is not intended to be inclusive with respect to terminology or eras of usage. Abbreviations: FCFC, fibroblast colony-forming cell; CFU-F, colony-forming unit-fibroblast; MSC, mesenchymal stromal cell; M, marrow; AT, adipose tissue; CB, cord blood; WJ, Wharton’s jelly. Created with BioRender.com.

The terminology used to describe MSCs has varied widely over the decades, and in general reflects changing paradigms related to their functional properties in vitro and mode of action in pre-clinical disease models (Figure 2). Friedenstein initially postulated that osteogenic cells in bone marrow were precursors of mechanocytes, a general term that includes cells derived from bone, cartilage, muscle, or connective tissue. He also coined the term FCFC to describe adhesive cells amenable to culture expansion and quantified their fibroblast colony-forming efficiency (CFE-F), which he defined as the ratio of colony number to the total number of cells plated. The term CFE-F was supplanted by CFU-F following the convention used in the hematopoiesis field, and over time, this activity-based assay was used interchangeably with MSCs as a descriptor. Friedenstein also used the terms stromal or reticular, fibroblast subtypes in bone marrow, to describe adhesive cells derived from FCFCs following culture expansion. By the 1980s, the term marrow stromal cells became widely adopted in the literature to describe adhesive marrow fibroblasts, which had been shown to express a multitude of adhesion molecules and cytokines/chemokines that supported the growth of hematopoietic cell lineages [24]. Consequently, marrow stromal cells were widely employed as feeder layers to establish long-term bone marrow cultures [25,26], a vital step in deciphering the nature of the HSC [24,27]. These findings explained Friedenstein’s observation that FCFCs could transfer the hematopoietic micro-environment to ectopic sites in vivo. Marrow stromal cells also possessed the ability to differentiate into adipocytes, chondrocytes, and osteoblasts in vitro, a feature that distinguished them from other sources of fibroblasts. As the latter garnered more attention in the field, the early 1990s saw the introduction of the term mesenchymal stem cell [28], which was postulated to reside at the apex of a mesengenic process that, via directed differentiation, yields determined cell types comprising connective tissues, including muscle, bone, and cartilage [29]. However, the use of this term drew the ire of key opinion leaders in the field since MSCs did not fit the conventional definition of bona fide stem cells [30], and efforts to retire the term gained significant traction in the following decades as research indicated that MSCs exert therapeutic effects via paracrine action and not by the replacement of damaged or diseased tissues via direct differentiation [31,32,33,34]. To quell the controversy, Caplan introduced the term medicinal signaling cells [31,35], a clever play on the MSC acronym, to reflect this new mode of action. Nevertheless, disputes over nomenclature continued, fueled mostly by arguments that the frequency of bona fide stem cells in MSC cultures is too low to be meaningful and that rigorous assays do not exist to quantify their self-renewal. While the former argument does not negate the existence of the MSC or its importance, Friedenstein’s ectopic osseous tissue formation assay has been replicated by various groups. For example, prospective isolation by FACS has identified progenitor populations from mouse [36,37] and human [38] bone marrow that exhibit varying potencies, including a population of PDPN^+^ CD146^−^CD73^+^CD164^+^ multi-potent progenitors capable of CFU-F formation in vitro, endochondrial ossification in vivo, and generation of hematopoiesis-supporting stroma. Importantly, these cells were also shown to regenerate heterotopic osseous tissue upon serial transplantation in vivo, thereby demonstrating their capacity for self-renewal [38]. Irrespective of these functional attributes, these cells are defined as progenitors, thereby reflecting the continued bias in the field regarding osteogenic stem cells. 

To provide further clarity to the field, the International Society of Cell & Gene Therapy (ISCT) [39] published a position paper recommending that bulk populations of adhesive fibroblasts be described as “mesenchymal stromal cells”. The authors were careful to stipulate that such populations are functionally heterogeneous and likely contain a small proportion of stem and/or progenitor cells. They also argued for the development of a matrix of phenotypic and functional assays to define the paracrine action of populations, including the secretion of trophic, pro-angiogenic, and immuno-modulatory mediators. Most recently, the ISCT proposed MSCs be defined by their tissue of origin, adopting a nomenclature system using abbreviations based on the International Society of Blood Transfusion 128 terminology model (Figure 2) [40]. This proposal reflects results from RNA-sequencing based studies showing human MSCs sourced from different tissues express a core set of genes that reflect their identity as well as differentially expressed gene subsets that are unique to their tissue or origin [41,42,43,44,45].

## 5. Mechanistic Link between Stem and Stromal Critical Quality Attributes

While efforts to reconcile MSC nomenclature are noteworthy, they reflect long-standing biases in the field. For example, under optimized conditions, more than 50% of plated adhesive fibroblasts possess CFU-F activity, and most are bi- or multi-potent, with respect to differentiation potential. Therefore, bulk MSC populations contain significant numbers of progenitors. To the purist, these are not self-renewing stem cells. However, identification by lineage tracing and prospective cell sorting of SSPCs in different skeletal compartments with varying differentiation and hematopoiesis-supporting capacities suggests rigid concepts about these populations may need revision, especially from an MSC-centric perspective. Additionally, while the paracrine functions of MSCs are thought to reside in the bulk stromal cell fraction, this compartment is also functionally heterogeneous and poorly defined. For example, the analysis of human MSC single cell-derived clones revealed secreted levels of nerve growth factor (NGF) varied 2000-fold, brain-derived neurotrophic factor (BDNF) varied 167-fold, interleukin 11 varied 265-fold, and stromal cell-derived factor 1α (SDF-1α) varied 16-fold between clones. Moreover, BDNF and NGF levels were highly correlated but not correlated with SDF1 [46] Consistent with these results, other studies have reported significant inter-donor differences in the ability of MSCs to induce IDO1 expression and suppress T cell proliferation in response to interferon-gamma treatment [47,48,49,50]. A recent study by Gao et al. [51] demonstrated that, in addition to promoting the maintenance of HSCs by secreting SCF, CXCL12, CSF1, and pleiotrophin, LepR ^+^ SSPCs also maintain bone marrow nerve fibers via the production of NGF, which, in turn, promotes hematopoietic and vascular regeneration. These results clearly demonstrate that bone marrow resident SSPCs simultaneously exhibit stem/progenitor and paracrine critical quality attributes, suggesting the “stem” vs. “stromal” cell dichotomy is oversimplified and probably incorrect. 

**Figure 3:**
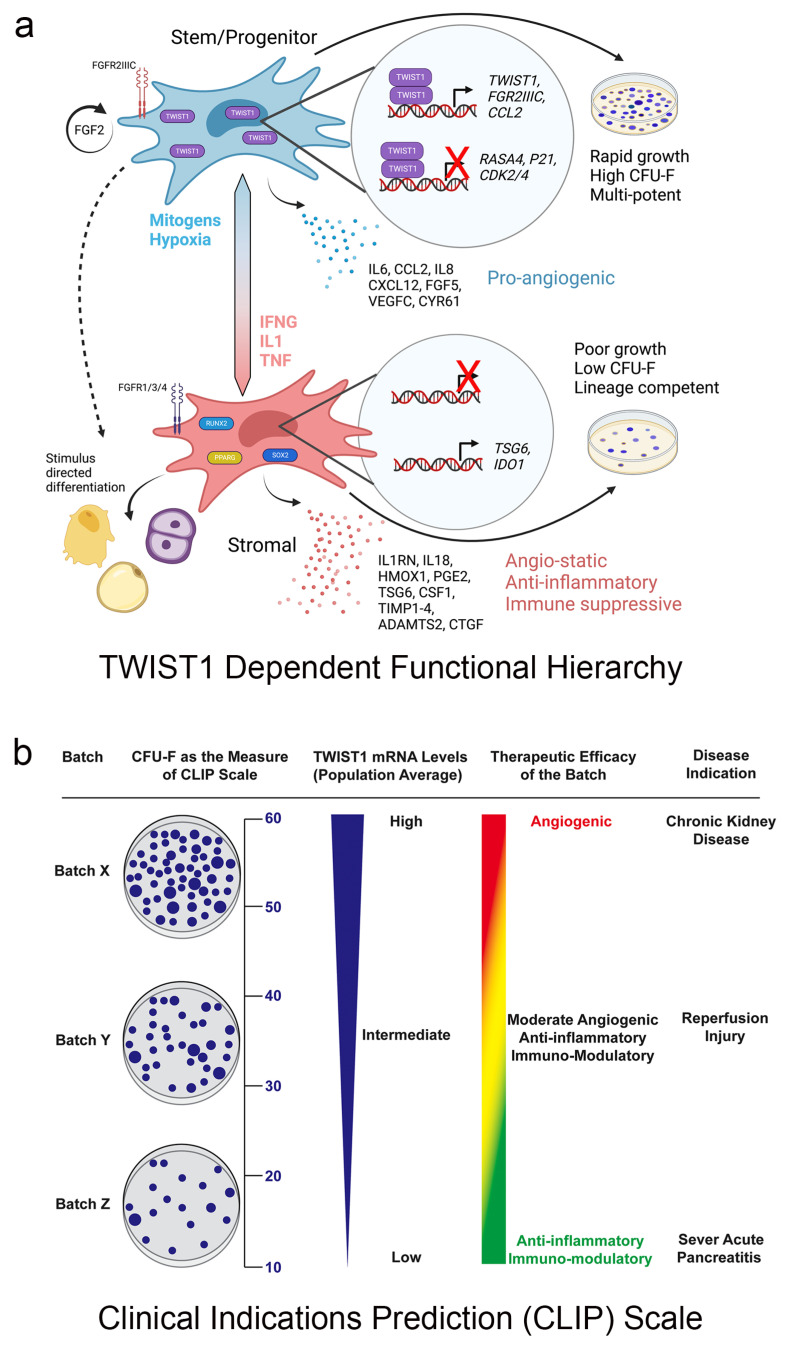
TWIST1 mechanistically links MSC stem/progenitor and paracrine functions and forms the basis of a Clinical Indications Prediction (CLIP) scale. (**a**) Schematic of a TWIST1 functional hierarchy where *TWIST1^Hi^* MSC are stem/progenitor-like and pro-angiogenic and *TWIST1^Low^* cells are stromal-like, anti-inflammatory, and immuno-modulatory. Agents promoting stem/progenitor or stromal phenotypes are indicated. Reprinted with permission from *Stem Cells* 2023; 41(5):444–452 [77]. (**b**) CLIP scale showing how CFU-F activity (left) is a surrogate for *TWIST1* mRNA levels and used to assign a “batch” to the proper disease indication. See text for more information. Reprinted with permission from *J Stem Cell Res Ther* 2016; 6:365 [53].

Recent data have also demonstrated a mechanistic link between MSC stem/progenitor and paracrine critical quality attributes. Specifically, we reported that MSCs from human bone marrow exhibit significant inter-donor differences in growth, viability, CFU-F activity, and tri-lineage differentiation and that population-averaged, normalized *TWIST1* and *FGFR2IIIC* levels predict donor-dependent differences in these quality attributes [52]. Mechanistic studies further showed that FGF2 induced *TWIST1* in a dose-dependent manner, and siRNA-mediated silencing of *TWIST1* induced the expression of CDKN1A, RASA4, *RUNX2, PPARG,* and *SOX4,* thereby linking TWIST1 to cell growth and differentiation [53]. The downregulation of *TWIST1* also repressed transcripts encoding proteins with pro-angiogenic activity while inducing those with angio-static, anti-inflammatory, and immuno-modulatory activity [52]. Subsequently, TWIST1 was shown to bind to the CCL2 and IDO1 promoters and induce and repress these genes via FGF2 and IFNG-dependent mechanisms, respectively. Activity-based assays further revealed that MSC pro-angiogenic activity correlated with *TWIST1* levels and was augmented by FGF2 and inhibited by IFNG, and that immuno-modulatory activity was inversely correlated with *TWIST1*, inhibited by FGF2, and augmented by IFNG [52]. These data clearly demonstrate that MSC stem/progenitor and paracrine activities are coordinately regulated, mechanistically linked via TWIST1, and specified hierarchically such that *TWIST1^Hi^* MSCs are stem/progenitor-like and pro-angiogenic, while *TWIST1^Low^* MSCs are stromal-like, anti-inflammatory, and immuno-modulatory (Figure 3a). Based on these data, we developed a Clinical Indications Prediction (CLIP) scale to forecast the potency of a given donor population based on *TWIST1* expression levels, and match populations to the appropriate disease indication or patient population (Figure 3b) [52,53]. In its present configuration, the CLIP scale assigns *TWIST1^Hi^* MSCs (batch X, >50 CFU-Fs) to ischemic disease indications, *TWIST1^Low^* cells (batch Z, <20 CFU-Fs) to immune/acute inflammatory diseases, and intermediate *TWIST1* cells (batch Y, 20–50 CFU-Fs) to diseases where both activities are beneficial. 

Most recently, we identified a high-value set of 143 unique TWIST1 targets in MSCs using a multi-omics approach [54], including TNF-stimulated gene/protein 6 (TSG6), a key mediator of MSC paracrine activity [55,56,57,58,59,60,61,62]. We also confirmed that TWIST1 directly repressed *TSG6* expression, and these mRNAs are inversely correlated in all MSC donors tested to date. Furthermore, we demonstrated that *TWIST1^Hi^* MSCs stimulated collagen invasion and tubule formation by HUVECs to a significantly greater extent than *TSG6^Hi^* MSCs, which are *TWIST1^Low^*, and that *TSG6^Hi^* MSCs were more potent in suppressing CD3^+^ T cell proliferation in vitro and preventing disease onset in an adoptive transfer model of Type 1 diabetes mellitus (T1D) compared to *TWIST1^Hi^* MSCs [63]. Lastly, we reported that *TWIST1* and *TSG6* levels are positively and negatively correlated, respectively, with the height of the MSC donor and confirmed that MSCs sourced from a tall (72 cm) vs. short (55 cm) statured donor exhibit dramatic differences in growth and CFU-F activity. 

A growing body of published research corroborates a mechanistic link between MSC stem/progenitor and paracrine functions. For example, FGF2, human platelet lysate, and hypoxia all positively impact MSC growth and pro-angiogneic activity [64,65,66,67,68], and both FGF2 and hypoxia also induce *TWIST1.* Conversely, IFNG, which is used to license MSC immuno-modulatory activity, suppresses TWIST1 and inhibits growth [52,69], and IFNG priming has been shown to convert MSCs from a pro-angiogenic to angio-static phenotype [70,71,72]. Other studies have shown that critical quality attributes conferring angiogenic activity onto MSCs are inversely correlated with those that predict immuno-modulatory activity and vice versa [50] and that MSCs cultured in human platelet lysate (hPL) exhibit enhanced growth and a reduced ability to inhibit allo-antigen-induced T-cell proliferation compared to those cultured in FBS-supplemented media [73]. Similarly, patients with steroid-refractory acute or chronic graft vs. host disease infused with MSCs expanded in hPL vs. FBS showed lower response rates [74,75]. Most recently, McKinnirey et al. [76] reported that female vs. male MSCs suppressed PBMNC proliferation to a greater extent in vitro, and this difference in activity was correlated with increased expression of IDO1, IL1RN, and PGE2. These findings challenge existing tenets that MSC potency is determined by the host injury micro-environment in vivo and that stem/progenitor functions are dispensable for cellular paracrine functions.

## 6. Future Perspective

After six decades, the meaning and significance of Friedenstein’s discoveries are still influencing the field of MSC research. While current research has uncovered potential new modes of MSC action, including efferocytosis, instant blood-mediated inflammatory reactions, and exosomes, the published literature is also converging on the viewpoint that MSC stem/progenitor and paracrine critical quality attributes are linked mechanistically and are systematized in populations. Nevertheless, recent efforts to build a consensus definition and reporting guidelines for MSCs [78] excluded characteristics such as population doubling rates and CFU-F activity despite their correlation with *TWIST1*, *TSG6,* and cell potency. This bias in reporting obfuscates ongoing efforts to critically interrogate MSC biology, which remains vitally important considering the poor success rate of experimental MSC-based therapies evaluated to date. Alternatively, building databases that link isolation/propagation protocols with basic biological characteristics and functional outcomes of cell-based activity assays and pre-clinical/clinical testing would represent a significant advance in the field, afford greater accountability, and provide training sets for machine/deep learning algorithms to aid in defining the most critical quality attributes that predict potency and clinical efficacy. Establishing a specific molecular signature for subpopulations of defined potency would further establish unique MSC fingerprints for different therapeutic applications. This holistic approach is progressive compared to previous efforts, such as establishing minimal criteria to define MSCs, which were used indiscriminately to the great detriment of the field. Because the CLIP scale employs quantifiable metrics that are sensitive to culture conditions, it represents a useful platform to optimize donor selection and manufacturing processes and quantify product potency prior to patient administration, a key first step in bringing continuity to the field and advancing MSC-based therapies toward regulatory approval. My team has also identified numerous genes that track with the expression of *TWIST1* and *TSG6* in MSCs, thereby establishing molecular fingerprints for populations with pro-angiogenic (*TWIST1*^Hi^) vs. anti-inflammatory and immuno-modulatory (*TSG6*^Hi^) populations. Defining the precise relationship between bone marrow resident and culture-expanded MSCs is also critically important, considering culture conditions markedly impact population heterogeneity and function, and many groups use proprietary media formulations for large-scale manufacturing. 

## Abbreviations

BDNF	Brain-derived neurotrophic factor
CCL2	C-C motif chemokine ligand 2
CDKN1A	Cyclin dependent kinase inhibitor 1A
CFE-F	Colony-forming efficiency
CFU-F	Colony-forming unit-fibroblast
CLIP.	Clinical indications prediction scale
CSF1	Colony stimulating factor 1
CXCL12	C-X-C motif chemokine ligand 12
EGF	Epidermal growth factor
FBS	Fetal bovine serum
FCFC	Fibroblast colony-forming cell
FGF	Fibroblast growth factor
FGFR2IIIC	Fibroblast growth factor receptor 2 IIIC
IFNG	Interferon gamma
hPL	Human platelet lysate
HSC	Hematopoietic stem cell
IDO1	Indoleamine 2,3-dioxygenase 1
ISCT	International Society of Cell & Gene Therapy
IL1RN	Interleukin 1 receptor antagonist
LepR	Leptin receptor
MSC	Mesenchymal stem cell
NGF	Nerve growth factor
PDGF	Platelet-derived growth factor
PGE2	Prostaglandin E synthase 2
PPARG	Peroxisome proliferator activated receptor Gamma
Rad	Radiation unit
RASA4	RAS P21 protein activator 4
RUNX2	RUNX family transcription factor 2
SCF	Stem cell factor
SDF-1α	Stromal cell-derived factor 1 alpha
SOX4	SRY-Box transcription factor 4
SSPC	Skeletal stem/progenitor cell
TGF-beta	Transforming growth factor beta
TSG6	TNF-stimulated gene/protein 6
TWIST1	Twist family BHLH transcription factor 1
